# Editorial: Tumor microenvironment and cancer therapy

**DOI:** 10.3389/fcell.2023.1290456

**Published:** 2023-09-22

**Authors:** Yi Xie, Jindong Xie, Pranav Gupta, Zhe-Sheng Chen, Shaoquan Zheng

**Affiliations:** ^1^ State Key Laboratory of Oncology in South China, Sun Yat-sen University Cancer Center, Guangzhou, China; ^2^ Department of Pharmaceutical Sciences, College of Pharmacy and Health Sciences, St. John’s University, New York, NY, United States; ^3^ Department of Breast Surgery, Breast Disease Center, The First Affiliated Hospital, Sun Yat-sen University, Guangzhou, China

**Keywords:** tumor microenvironment, cancer therapy, oncology, immune, bioinformatics

The tumor microenvironment (TME), an integral part of tumor, is comprised of all the non-cancerous host cells in the tumor, including fibroblasts, endothelial cells, neurons, adipocytes, adaptive, and innate immune cells. It also consists of non-cellular components, including the extracellular matrix (ECM) and soluble products such as chemokines, cytokines, growth factors, and extracellular vesicles (Bejarano et al., 2021). Accumulating evidence indicates that cellular and acellular constituents in TME play a pivotal role in reprogramming tumor initiation, growth, invasion, metastasis and response to therapies (Park et al., 2023; Zhang et al., 2023). Although considerable progress has been made in TME-targeted cancer therapy, such as immune checkpoint blockade (ICB), chimeric antigen receptor (CAR) T-cell therapy and oncolytic virus therapy, the clinical efficacy of therapeutic strategies targeting TME, especially the specific cells or pathways of TME, remains unsatisfactory. It is imperative to identify new targets and biomarkers that could improve the clinical efficacy and precision in specific patients. The current Research Topic, “*Tumor microenvironment and cancer therapy*”, brings together researchers at the forefront of the much-anticipated field with a series of authoritative reviews and exciting original articles to provide a timely update on our current understanding of biology of the TME and therapies to target it.

Anticancer drug resistance presents a substantial barrier to cancer treatment (Sun et al., 2022). Although many approaches have been employed to study drug resistance, its mechanisms still remain poorly understood. Si et al. provided an overview that microRNAs, a large family of post-transcriptional regulators of gene expression, play a significant role in regulating the resistance of anthracyclines in breast cancer therapy and demonstrate the potential as prognostic biomarkers. TME has emerged as a critical factor in both drug efficacy and chemoresistance in recent years, impelling the development of novel therapeutic strategies. Chen et al. submitted a comprehensive review on the roles of hypoxia in the tumor microenvironment and targeted therapy. They observed immunosuppression and metabolic reprogramming in TME under the condition of hypoxia. Immunosuppressive cells such as MDSCs, TAM and Tregs were recruited to the hypoxic parts to facilitate the escape of tumor cells and resistance to anticancer drug. The metabolic reprogramming of tumor cells was conducive to obtaining energy in a hypoxic environment while maintaining an acidic microenvironment. Apart from chemotherapy resistance, immunotherapy resistance also attracts much attention. Yu et al. identified nine hub genes (*COL1A1, COL1A2, COL3A1, COL5A1, COL5A2, COL6A3, EMILIN1, MMP2,* and *THY1*) as potential predictors for implying cancer stemness and immunotherapy resistance to lung adenocarcinoma (LUAD) by Weighted Gene Co-Expression Networks Analysis (WGCNA) and protein-protein interaction (PPI) network analysis and found that *THY1* might serve as a prognostic marker.

Although immune checkpoint blockade has brought new hope for many cancers, it has not yet provided substantial benefit to patients with glioma. Utilizing a range of analyses involving protein networks, survival, clinical correlation and function, Zhou et al. demonstrated that the levels of specific ligands (PDL1/PDL2 and CD80/CD86) were significantly higher in the subgroup of glioma patients with high expression levels of *HSAP6*, which might provide new insights into immunotherapy for glioma patients with a deeper understanding of the correlation between *HSPA6* and immunity. Additionally, the ECM is a dynamic and complex meshwork consisting of various multi-domain macromolecules that are continually synthesized and secreted by surrounding cells. On this subject, Fu et al. emphasized that Tenascin C (TNC), a non-structural protein in the ECM, was implicated in the malignant progression of glioma, including proliferation, neovascularization, invasiveness, and immunomodulation. Thorough research on TNC, particularly on the different domains and critical targets of the signaling pathway, may provide new therapeutic strategies for glioma treatment.

Metastasis, a primary cause of death in patients with malignancies and a challenge for cancer therapy, is promoted by intrinsic changes in both tumor and non-malignant cells in the TME. Yan et al. focused their study on the roles of tumor-associated neutrophils (TANs) in tumor metastasis, which can be a potential target of cancer treatment. In another study, Tan et al. analyzed the role of *circPLK1* in cancer cell proliferation and metastasis. They demonstrated that *circPLK1* could sponge miR-186, which upregulated the downstream *DNMT3A* expression and triggered the DNA hypermethylation of *APCDD1* promoter, leading to *APCDD1* downregulation and promoting the cell invasion and metastasis in osteosarcoma.

Prognostic biomarkers for various types of cancer were also investigated based on the signature of TME. Li et al. identified *OAS3* as a prognostic and immunotherapeutic biomarker that was associated with unfavorable survival outcomes. Yu et al. investigated the role of *PUDP* in hepatocellular carcinoma (HCC) and showed that *PUDP* was highly expressed in most cancers and high expression of *PUDP* indicated a poor prognosis and lower response rates to immunotherapy in HCC.

Fibroblasts play an essential role in TME. Gao et al. discovered the underlying mechanism of keloid scars (KS) recurrence. The authors focused on FAP + fibroblasts, which are crucial for KS recurrence after surgical resection and radiation therapy. Their findings revealed that irradiation promoted cell cycle progression in FAP + fibroblasts due to the ATP production, upregulation of Cyclin D1 and downregulation of the CKIs p53, p21, and p16, which was associated with cell proliferation and delayed cellular senescence.

In conclusion, TME is a complicated and highly heterogeneous circumstance that can affect almost every aspect of cancer biology. It brings therapeutic targets for cancer treatment, which still needs further discussion and validation. In addition, TME is the inevitable part of tumor bulk and understanding the complexity of it lays a solid foundation for the development of more effective biomarkers and therapies for cancer. We hope that the findings in this Research Topic can inspire further innovations and look forward to an exciting future in this field.
